# To Detect Horseflesh from Beef

**Published:** 1898-09

**Authors:** 


To Detect Horseflesh from Beef. M. Humbert, through the *Reveil de Medicine Veterinaire*, tells us that chemical tests will determine whether a given specimen of meat is from the horse or from the flesh of any other animal usually used for food. Fifty grammes of meat are boiled for one hour in 200 grammes of water, and then the decoction is set aside to cool. When cool, nitric acid in the proportion of 5 per cent. is added, and into this is dropped, drop by drop, some of Gram's iodo-ioduret solution, or in its place a solution of iodized water which has been well iodized by the aid of heat; if horseflesh is present, there will appear a deep violet-red circle. Neither beef, veal, mutton, nor pork will furnish the same reaction.—*Popular Science News.*

